# Safety and efficacy of fruquintinib‐based therapy in patients with advanced or metastatic sarcoma

**DOI:** 10.1002/cam4.7438

**Published:** 2024-07-05

**Authors:** Chenliang Zhou, Guowei Qian, Yonggang Wang, Hongtao Li, Zan Shen, Shuier Zheng

**Affiliations:** ^1^ Department of Oncology Shanghai Sixth People's Hospital Affiliated to Shanghai Jiao Tong University School of Medicine Shanghai China

**Keywords:** angiogenesis, fruquintinib, multi‐target tyrosine kinase inhibitors, progression‐free survival, sarcoma

## Abstract

**Background:**

The purpose of this study was to evaluate the efficacy and safety of fruquintinib‐based therapy as a salvage therapy for patients with advanced or metastatic sarcoma, including soft tissue sarcoma (STS) and bone sarcoma.

**Methods:**

Patients with advanced or metastatic sarcoma were divided into two groups. One group received fruquintinib monotherapy, while the other received fruquintinib combined therapy. Safety and efficacy of fruquintinib‐based therapy were recorded and reviewed retrospectively, including progression‐free survival (PFS), overall response rate (ORR), and adverse events (AEs).

**Results:**

Between August 2021 and December 2022, 38 sarcoma patients were retrospectively included. A total of 14 patients received fruquintinib alone (including 6 STS and 8 bone sarcoma), while 24 were treated with fruquintinib combined therapy (including 2 STS and 22 bone sarcoma). The median follow‐up was 10.2 months (95% CI, 6.4–11.5). For the entire population, the median PFS was 8.0 months (95% CI, 5.5–13.0). The ORR was 13.1%, while the disease control rate (DCR) was 86.8%. The univariate analysis showed that radiotherapy history (HR, 4.56; 95% CI, 1.70–12.24; *p* = 0.003), bone sarcoma (HR, 0.34; 95% CI, 0.14–0.87; *p* = 0.024), and treatment method of fruquintinib (HR, 0.36; 95% CI, 0.15–0.85; *p* = 0.021) were significantly associated with PFS. The multivariate analysis showed that patients without radiotherapy history were associated with a better PFS (HR, 3.71; 95% CI: 1.31–10.55; *p* = 0.014) than patients with radiotherapy history. Patients in combination group reported pneumothorax (8.3%), leukopenia (33.3%), thrombocytopenia (12.5%), diarrhea (4.2%), and anemia (4.2%) as the most frequent grade 3 or higher treatment‐emergent AEs (TEAEs), while there was no severe TEAEs occurred in the monotherapy group.

**Conclusions:**

Fruquintinib‐based therapy displayed an optimal tumor control and an acceptable safety profile in patients with advanced or metastatic sarcoma.

## 1 BACKGROUND

Sarcomas are a group of solid malignant tumors with over 70 histopathological subgroups originating from mesenchymal tissues.^1^, ^2^, ^3^ Sarcoma is a rare disease that accounts for 1% and 15% of solid malignant tumors in adults and pediatric patients, respectively.^1^ Based on the different origin sites, sarcomas can be classified into soft tissue sarcomas (STS) and bone sarcomas.

Surgery is the primary therapy for sarcoma treatment.^1^, ^2^, ^4^, ^5^ Nevertheless, some are diagnosed as metastatic at presentation or after surgery.^6^ Chemotherapy is recommended as first‐line treatment for these patients.^7^, ^8^, ^9^ However, the response rates to chemotherapy are only 14%–48%.^10^ Therefore, it is imperative to explore effective treatments for advanced or metastatic sarcoma.

Vascular endothelial growth factor (VEGF) and its receptor, the VEGF receptor (VEGFR), are implicated in the chronic angiogenesis of sarcomas, playing a critical role in cancer development, invasion, and metastasis.^11^, ^12^, ^13^ Consequently, targeting tumor angiogenesis may be a crucial therapeutic approach against cancer.^14^, ^15^, ^16^ Clinically developed multi‐target tyrosine kinase inhibitors (TKIs) targeting VEGFR have demonstrated efficacy in the treatment of various malignancies.^17^, ^18^, ^19^ Several previous studies have indicated that multi‐target TKIs have anti‐tumor effect in sarcoma.^14^, ^20^ According to the results of the PALETTE phase III clinical trial,^21^ pazopanib is the first multi‐target TKI approved by the Food and Drug Administration (FDA) for patients with advanced and metastatic non‐adipocytic STS with previously chemotherapy. In the cohort of 369 sarcoma patients treated with pazopanib,^22^ the median progression‐free survival (mPFS) was 4.6 months, and the median overall survival (mOS) was 12.5 months. Anlotinib was used to treat refractory or metastatic STS, which exhibited mPFS of 5.6 months, overall response rate (ORR) of 13% (95% CI, 7.6–17), and disease control rate (DCR) of 74% (95% CI, 66–80).^15^ Additionally, anlotinib showed a 3‐month PFS rate of 75.86% and mPFS of 4.8 months in patients with advanced osteosarcoma who had previously received intensive treatment.^14^ Apatinib demonstrated mPFS of 7.88 months, ORR of 8.88% (4/45) and DCR of 88.89% (40/45) in the treatment of advanced sarcoma as second‐line or subsequent treatment.^23^ However, apatinib was associated with higher incidences of adverse events (AEs), with dose‐reduction among 21 (46.67%) patients.

Fruquintinib, a multi‐target TKI, targets tumor angiogenesis associated with tumor growth. It is an effective and selective small‐molecule inhibitor of VEGFR‐1, ‐2, and ‐3.^24^, ^25^ The China National Medical Products Administration and the FDA has granted authorization for anti‐tumor treatment of fruquintinib as a third line or above in metastatic colorectal cancer.^26^

In this retrospective study, patients received either fruquintinib monotherapy or a combination treatment including fruquintinib. The purpose of the study was to evaluate the efficacy and safety of fruquintinib‐based therapy in patients with advanced or metastatic sarcoma.

## 2 MATERIALS AND METHODS

### 2.1 Patients eligibility

The retrospective study was conducted at Shanghai Sixth People's Hospital Affiliated to Shanghai Jiao Tong University School of Medicine. This study involving participants was conducted in accordance with the Declaration of Helsinki. It was approved by the Institutional Review Board of Shanghai Sixth People's Hospital Affiliated to Shanghai Jiao Tong University School of Medicine (2022‐KY‐088K). All patients were enrolled between August 2021 and December 2022.

The eligibility criteria were as follows: (1) pathologically confirmed sarcoma, subsequently diagnosed as advanced or metastatic through imaging; (2) at least one assessable lesion based on Response Evaluation Criteria in Solid Tumors (RECIST version 1.1); (3) available clinicopathological variables. Baseline clinicopathological variables for each patient included: age, sex, clinical stage, primary tumor site, histology, Eastern Cooperative Oncology Group (ECOG) performance status, metastasis sites, and treatment history. The follow‐up period of this study was extended to April 30, 2023.

### 2.2 Treatment methods

Patients were divided into two groups. One group received a combination of fruquintinib with chemotherapy, targeted therapy, or immune checkpoint inhibitors (ICIs), while the other group received fruquintinib monotherapy. On days 1–21 of a 28‐day cycle, patients received a once‐daily oral dosage of 4 mg of fruquintinib for patients less than 18‐years‐old, or 5 mg if they were 18‐years‐old or older. For those experiencing intolerable AEs, the fruquintinib dose was reduced in 1 mg increments until the toxicity was tolerable. The utilization of fruquintinib was same in both the combination group and the monotherapy group. The chemotherapy regimens included gemcitabine plus docetaxel (gemcitabine = 675–1000 mg/m^2^ on days 1 and 8, docetaxel = 75 mg/m^2^ on day 8 every 21 days), ifosfamide plus etoposide (ifosfamide 2–2.5 g/m^2^ on days 1–4, etoposide 100–120 mg/m^2^ on days 1–4), irinotecan (50 mg/m^2^ on days 1–5) and pirarubicin (40–50 mg/m^2^ on day 1). The therapy of targeted therapy included abemaciclib (150 mg twice daily) and trametinib (2 mg once daily). Envafolimab is a PD‐L1 inhibitor, approved in China, administered at 400 mg every 21 days for adult patients with advanced MSI‐H or dMMR solid tumors.

Computed tomography or magnetic resonance imaging was used to evaluate the tumor response after treatment, according to RECIST (version 1.1). The ORR was determined by the percentage of patients with the best overall response (confirmatory imaging was not conducted for all 38 patients) of complete response (CR) or partial response (PR). The percentage of patients with the best tumor response of CR, PR, or stable disease (SD) was determined as DCR. PFS was calculated from fruquintinib initiation to disease progression or death from any cause.

Treatment continued until intolerable toxicity, the appearance of progressive disease (PD) or curative surgery following the resection of metastasis or recurrence tumors. Adverse Events (AEs) were recoded according to the National Cancer Institute Common Terminology Criteria for AEs (version 5.0).

### 2.3 Statistical analysis

Quantitative data were presented as medians (ranges) or patient counts (percentages). The Kaplan–Meier method was employed to calculate PFS with a 95% confidence interval (CI). The reverse Kaplan–Meier method was used to calculate the median follow‐up time. The statistical analysis and the waterfall plots were performed using StataCorp.2021. (Stata Statistical Software. Release 17.) and R version 4.2.2 (R Core Team (2023).).

## 3 RESULTS

### 3.1 Patient characteristics

In total, 38 sarcoma patients were enrolled, including 8 with STS and 30 with bone sarcoma. Fourteen patients received fruquintinib monotherapy (including 6 with STS and 8 with bone sarcoma), while 24 patients received fruquintinib combined therapy (including 2 with STS and 22 with bone sarcoma). Of these, 18 (75%), 4 (16.7%), and 5 (8.3%) patients were combined with chemotherapy, chemotherapy plus ICIs, and targeted therapy, respectively.

Among the patients who received chemotherapy, 14 patients received a combination of gemcitabine plus docetaxel, and 8 patients received other regimens. Two patients received fruquintinib in combination with abemaciclib or trametinib, respectively, based on the results of next‐generation sequencing (NGS) analysis of tumor tissues.

The demographics and baseline characteristics of enrolled patients are presented in Table 1. The most prevalent histological subtypes were osteosarcoma (25 patients, 65.8%) among bone sarcoma patients and undifferentiated pleomorphic sarcoma (UPS; 3 patients, 7.9%) in STS patients. Almost all the patients (92.1%) were in stage IV disease. 32 (84.2%) had lung metastases, 14 (36.8%) had bone metastases, 6 (15.8%) had pleural metastases, and 18 (47.4%) had metastases at other sites. All the patients has received previous lines of anti‐tumor therapy before fruquintinib with a median of 2 treatment lines. (Table 1).

**TABLE 1:** Baseline characteristics of patients.

Characteristics	Number of patients	Percentage (%)
Age, years		
Mean age	20	–
Range	8–71	–
Sex		
Male	22	57.9
Female	16	42.1
Clinical stage		
IIIB	3	7.9
IV	35	92.1
Location		
Limb	27	71.1
Trunk	6	15.8
Maxillofacial	5	13.2
Histology		
Bone	30	78.9
Osteosarcoma	25	65.8
Ewing sarcoma	3	7.9
Mesenchymal chondrosarcoma	1	2.6
Malignant transformation of giant cell tumor	1	2.6
Soft tissue	8	21.1
Pleomorphic rhabdomyosarcoma	1	2.6
Undifferentiated pleomorphic sarcoma	3	7.9
High‐grade undifferentiated spindle cell sarcoma	1	2.6
Leiomyosarcoma	1	2.6
Fibrosarcoma	1	2.6
Ewing sarcoma	1	2.6
ECOG PS score		
0–1	31	81.6
2	7	18.4
Metastasis site		
Lung	32	84.2
Bone	14	36.8
Pleura	6	15.8
Others	18	47.4
Radiotherapy history		
Yes	9	23.7
No	29	76.3
Surgery history		
Yes	31	81.6
No	7	18.4
Chemotherapy history		
Yes	38	100
No	0	0
Multi‐target TKIs therapy history		
Yes	14	36.8
No	24	63.2
Treatment lines of fruquintinib		
Second line	17	44.7
Third line or more than third line	21	55.3
Treatment pattern		
Fruquintinib alone	14	36.8
Fruquintinib + chemotherapy	18	47.4
Fruquintinib + chemotherapy + ICIs	4	10.5
Fruquintinib + targeted therapy	2	5.3

Abbreviations: ECOG PS score, ECOG performance status score; ICIs, Immune checkpoint inhibitors; Multi‐target TKIs, Multi‐target tyrosine kinase inhibitors.

### 3.2 Efficacy

**FIGURE 1:**
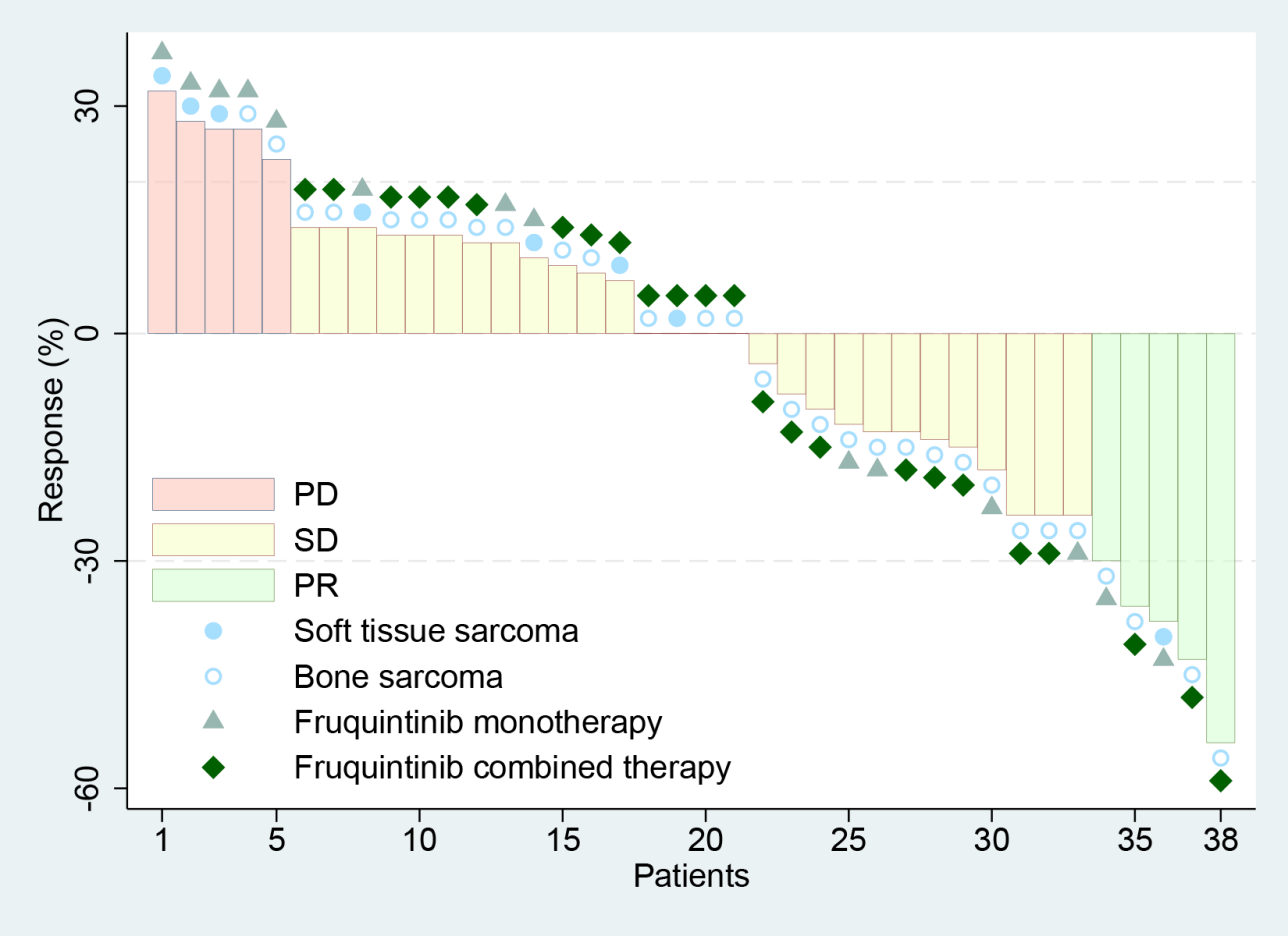
Waterfall plot of the best percentage changes for the sum of target lesion diameters after fruquintinib‐based treatment are shown for individual patients with best objective response per RECIST version 1.1 as indicate. PD, progressive disease; PR, partial response; SD, stable disease.

Objective response was documented in 5 (13.1%) of 38 patients, including 2 patients receiving fruquintinib monotherapy group (1 STS and 1 bone sarcoma) and 3 patients receiving fruquintinib combined therapy (3 bone sarcoma). No CR was observed (Figure 1). Twenty‐eight patients (73.7%) exhibited SD as the best overall response, including 12 bone sarcoma patients who experienced tumor shrinkage, yielding a DCR of 86.8%. Among these, 9 patients receiving fruquintinib monotherapy group (including 3 STS and 6 bone sarcoma) and 24 patients receiving fruquintinib combined therapy (including 2 STS and 22 bone sarcoma) achieved SD or PR (Figure 1).

**FIGURE 2:**
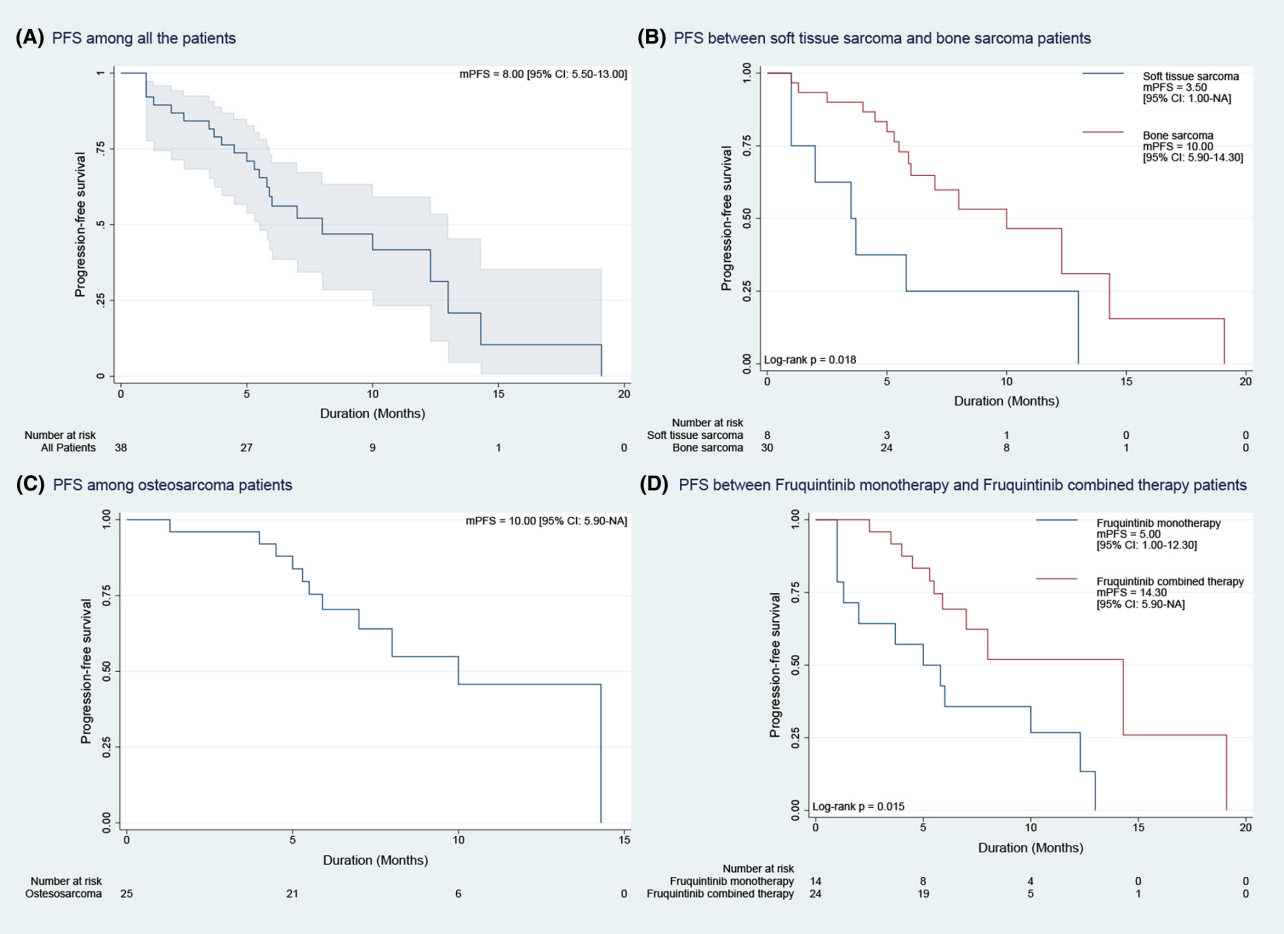
The Kaplan–Meier curve of median progression‐free survival (mPFS). (A) PFS among all the patients. (B) PFS between soft tissue sarcoma and bone sarcoma patients. (C) PFS among osteosarcoma patients. (D) PFS between Fruquintinib monotherapy and Fruquintinib combined therapy patients.

The median follow‐up time was 10.2 months (95% CI, 6.4–11.5). For the entire population, the mPFS was 8.0 months (95% CI, 5.5–13.0) (Figure 2A). Among patients with STS and bone sarcoma, the mPFS was 3.5 months (95% CI, 1.0–NA) and 10.0 months (95% CI, 5.9–14.3), respectively (Figure 2B). Moreover, mPFS were 10.0 months (95% CI, 5.9–NA) in patients with osteosarcoma, the most prevalent histological subtypes (Figure 2C). Additionally, the mPFS for the monotherapy group was 5.0 months (95% CI, 1.0–12.3), while the mPFS for the combination group was 14.3 months (95% CI, 5.9–NA) (Figure 2D).

**FIGURE 3:**
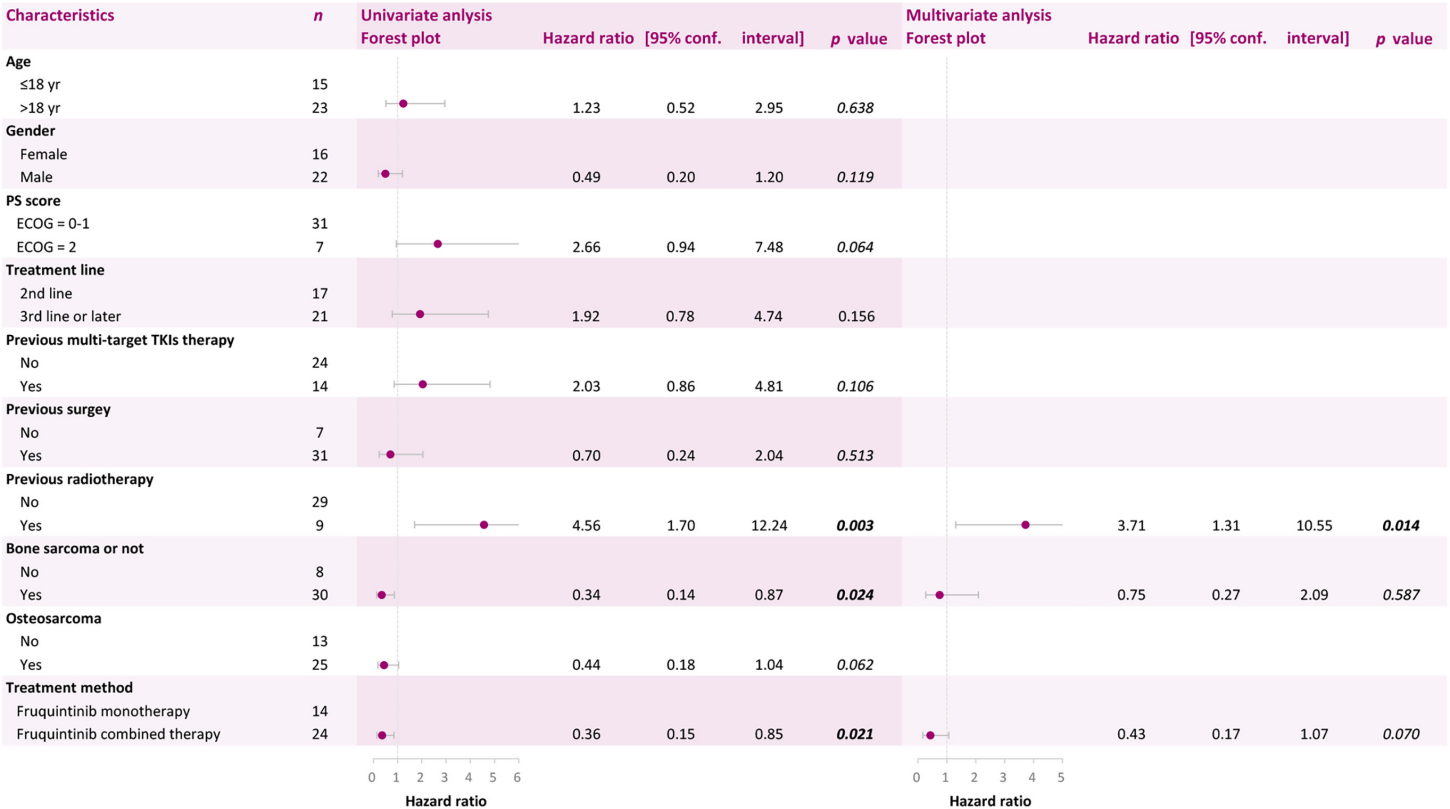
Univariate and multivariate Cox regression analysis demonstrated that the related characteristics are the prognosis of patients. PS score, performance status score.

The univariate analysis showed that a history of radiotherapy (hazard ratio [HR], 4.56; 95% CI, 1.70–12.24; *p* = 0.003), bone sarcoma (HR, 0.34; 95% CI, 0.14–0.87; *p* = 0.024), and treatment method of fruquintinib (HR, 0.36; 95% CI, 0.15–0.85; *p* = 0.021) were significantly associated with PFS. Results of the multivariate analysis demonstrated that a history of radiotherapy was an independent factor influencing PFS. Patients without previous radiotherapy had better PFS (HR, 3.71; 95% CI, 1.31–10.55; *p* = 0.014) compared to patients with such a history (Figure 3).

Moreover, 11 osteosarcoma patients proceeded surgery of metastasis or recurrence tumors following fruquintinib‐based therapy. Of these surgeries, 10 patients achieved R0 resection and 1 patient had R1 resection. Furthermore, three patients achieved a tumor necrosis rate of 90%. As of the data cutoff date, five patients remained disease‐free for more than 5 months (Table 2).

**TABLE 2:** Clinical characteristics of osteosarcoma patients proceeded surgery of metastasis or recurrence tumors after fruquintinib‐based therapy.

Patient	Treatment pattern	Metastasis site before surgery	Surgery site	Surgery type	Surgical margin	Disease‐free survival (months)	Tumor necrosis rate
1	Fruquintinib + chemotherapy	Pulmonary	Pulmonary metastasis	Curative surgery	R0	>8.3	–
2	Fruquintinib + chemotherapy	Pulmonary	Pulmonary metastasis	Curative surgery	R0	1	–
3	Fruquintinib + chemotherapy + ICIs	Pulmonary, bone	Bone metastasis	Palliative surgery	R0	–	40%
4	Fruquintinib	Pulmonary	Pulmonary metastasis	Palliative surgery	R0	–	–
5	Fruquintinib + chemotherapy	Pulmonary	Pulmonary metastasis	Curative surgery	R0	>5.5	–
6	Fruquintinib + chemotherapy	Pulmonary	Recurrence tumor	Palliative surgery	R0	–	–
7	Fruquintinib + chemotherapy	Pulmonary	Recurrence tumor	Palliative surgery	R0	–	50%
8	Fruquintinib + chemotherapy	Pulmonary, bone	Recurrence tumor	Palliative Surgery	R1	–	90%
9	Fruquintinib + chemotherapy	–	Recurrence tumor	Curative surgery	R0	>6.2	–
10	Fruquintinib + chemotherapy	–	Recurrence tumor	Curative surgery	R0	>5.2	90%
11	Fruquintinib + chemotherapy	Bone	Bone metastasis	Curative surgery	R0	>6.2	90%

Abbreviation: ICIs, immune checkpoint inhibitors.

### 3.3 Safety

Treatment‐emergent adverse events (TEAEs) are presented in Table 3. Hypertension (21.4%) was the most common any‐grade TEAEs observed in the fruquintinib monotherapy group, whereas leukopenia (70.8%) and thrombocytopenia (70.8%) were the most common any‐grade TEAEs in the combination group. Patients in the combination group reported pneumothorax (8.3%), leukopenia (33.3%), thrombocytopenia (12.5%), diarrhea (4.2%), and anemia (4.2%) as the most frequent grade 3/4 TEAEs. The combination group experienced some severe TEAEs, while there was no severe TEAEs occurred in the monotherapy group. No death were reported in relation to anti‐tumor treatment. Although three patients in the combination group experienced dose reductions due to the drug toxicity of pneumothorax or diarrhea, they were able to tolerate fruquintinib at the reduced doses of 3 or 4 mg.

**TABLE 3:** Treatment‐emergent adverse events during the treatment period.

Toxicity	The monotherapy group *N* = 14			The combination group *N* = 24		
	Grade 1–2 *N* (%)	Grade 3–4 *N* (%)	All grades *N* (%)	Grade 1–2 *N* (%)	Grade 3–4 *N* (%)	All grades *N* (%)
Pneumothorax	2 (14.3)	0	2 (14.3)	0	2 (8.3)	2 (8.3)
Fatigue	1 (7.1)	0	1 (7.1)	0	0	0
Nasal hemorrhage	1 (7.1)	0	1 (7.1)	0	0	0
Hemoptysis	1 (7.1)	0	1 (7.1)	0	0	0
Delayed wound healing	0	0	0	1 (4.2)	0	1 (4.2)
Rash	0	0	0	3 (12.5)	0	3 (12.5)
Leukopenia	0	0	0	9 (37.5)	8 (33.3)	17 (70.8)
Thrombocytopenia	0	0	0	14 (58.3)	3 (12.5)	17 (70.8)
Anemia	0	0	0	7 (29.2)	1 (4.2)	8 (33.3)
Nausea/vomiting	0	0	0	7 (29.2)	0	7 (29.2)
Diarrhea	0	0	0	1 (4.2)	0	1 (4.2)
Hypertension	3 (21.4)	0	3 (21.4)	2 (8.3)	0	2 (8.3)
Hand‐foot skin reaction	1 (7.1)	0	1 (7.1)	2 (8.3)	0	2 (8.3)
ALT elevation	2 (14.3)	0	2 (14.3)	10 (41.7)	0	10 (41.7)
AST elevation	2 (14.3)	0	2 (14.3)	10 (41.7)	0	10 (41.7)
Hypothyroidism	1 (7.1)	0	1 (7.1)	3 (12.5)	0	3 (12.5)
Proteinuria	1 (7.1)	0	1 (7.1)	1 (4.2)	0	1 (4.2)
Palpitation	1 (7.1)	0	1 (7.1)	0	0	0
Oral ulcer	2 (14.3)	0	2 (14.3)	0	0	0

Abbreviations: ALT, alanine transaminase; AST, aspartate transaminase.

## 4 DISCUSSION

The main therapeutic goals for patients with advanced or metastatic sarcoma are optimal tumor control and an acceptable safety profile over an extended period. Pathological angiogenesis is commonly in advanced or metastatic sarcoma, and plays a crucial role in cancer development, invasion, and metastasis.^12^, ^13^ Thus, blockade of tumor angiogenesis has been recognized as a significant anti‐tumor strategy for sarcoma. Numerous researches have shown the effectiveness of multi‐target TKIs in patients with advanced or metastatic sarcoma,^11^, ^14^, ^20^ but few have demonstrated the effectiveness of fruquintinib. These medications are multi‐target TKIs, but they may have varying anti‐tumor effects and toxicities in advanced or metastatic sarcoma. Moreover, fruquintinib, an effective and selective small‐molecule inhibitor of VEGFR‐1, ‐2, and ‐3, has shown a favorable tumor control and safety profile in malignant tumors.^25^, ^27^

In this study, patients with advanced or metastatic sarcoma received fruquintinib monotherapy or combination therapy. The ORR was 13.1%, while the DCR reached 86.8%. Notably, the mPFS was 10.0 months in patients with osteosarcoma, the most prevalent histological subtypes. Additionally, the mPFS for the combination group was 14.3 months. Therefore, fruquintinib‐based therapy, especially for the fruquintinib combination treatment or treatment in patients with bone sarcoma, has led to significant advances.

In a report by Ding X and his colleagues, fruquintinib was demonstrated as a petential third‐ or further‐line treatment for patients with advanced sarcoma. The mPFS for the fruquintinib‐based group and the control group were 4.8 versus 1.4 months (*p* < 0.001), respectively.^28^ Anlotinib showed a mPFS of 4.8 months in patients with advanced osteosarcoma who had previously received intensive treatment.^14^ Cabozantinib was used to treat advanced osteosarcoma with no limit on the number of previous lines of treatment. The outcomes revealed that ORR was 12%. Additionally, mPFS was 6.7 months and mOS was 10.6 months in this phase 2 trial.^20^ Our study demonstrated that fruquintinib is effective in the second‐ or further‐line therapy of bone sarcoma, seemingly showing a longer mPFS than other studies in osteosarcoma. It seems that the earlier treatment lines before fruquintinib and better performance scores may lead to the difference between the therapeutic effect of fruquintinib and the other multi‐target TKIs. A previous study found that 34.6% of patients with advanced STS could still benefit from other multi‐target TKIs after failing treatment with multiple TKIs. Among these patients, who received anlotinib, lenvatinib, apatinib, pazopanib, axitinib, or regorafenib as TKI rechallenge, the mOS was 11.7 months and the mPFS was 3.3 months.^29^ As a result of our study, 8 STS patients received fruquintinib after anlotinib failure, achieving a mPFS of 3.5 months, which still showed promising results. This finding suggests the potential for sequential targeted therapy to unveil new opportunities for treating STS.

In patient‐derived xenograft models, the combination of fruquintinib and chemotherapy showed a promising anti‐tumor activity.^24^ Through the pre‐clinical and clinical studies, fruquintinib and ICIs have also shown synergistically anti‐tumor activity.^30^ The mPFS of fruquintinib combined therapy in our study was seemingly much better (mPFS = 14.3 months, 95% CI, 5.9–NA) than TAG (docetaxel, bevacizumab, mPFS = 3.6 months, 95% CI, 2.0–7.6) for very high‐risk sarcoma in adolescents and young adults reported in a retrospective study.^31^ Moreover, fruquintinib combined therapy was likely more effective than sorafenib therapy combined with everolimus, which did not reach the prespecified target of 6 months of PFS of 50% or greater and had severe toxicity.^32^ However, further research is essential to confirm the superiority of fruquintinib‐based combined therapy over other combined therapies.

The previous studies have already revealed osteosarcoma patients who undergo curative surgery and chemotherapy have better survival rates than those whose metastases cannot be removed.^33^, ^34^, ^35^ Patients with tumor necrosis rate exceeding 90% after neoadjuvant chemotherapy frequently have better outcomes than their counterparts.^36^, ^37^ However, few studies have demonstrated the effectiveness of multi‐target TKIs as tumor conversion therapy in treating advanced or metastatic osteosarcoma. In a clinical study, the combination of anlotinib and chemotherapy followed by surgery increased the rate of tumor regression, surgical conversion and R0 resection, with an acceptable safety profile. This regimen was demonstrated as an optional treatment for patients with unresectable STS.^38^ In a real‐world study, 11 osteosarcoma patients underwent surgery after fruquintinib‐based therapy, with a conversion rate of 29%. These findings revealed that fruquintinib‐based therapy has favorable efficacy as tumor conversion therapy.

The univariate analysis showed that radiotherapy history, bone sarcoma, and the treatment method of fruquintinib were significantly associated with PFS. Furthermore, we observed previous radiotherapy was an independent factor influencing PFS through multivariate analysis. Specifically, radiotherapy was conducted to relieve pain or the patients with brain metastases or pathological fractures. Thus, patients without previous radiotherapy were associated with a better PFS than patients with previous radiotherapy in our study.

Most patients tolerated fruquintinib well in this real‐world study. The combination group experienced more TEAEs than the monotherapy group. TEAEs experienced by the combination group patients mainly associated with chemotherapy. The most frequent grade 3/4 TEAEs in the combination group were all related to myelosuppression caused by chemotherapy. Therefore, the therapies were conducted to improve myelosuppression, including G‐CSF, thrombopoietin, IL‐11 and erythropoietin. The therapies of closed thoracic drainage and oxygen inhalation were administered to the patients with severe pneumothorax. There was no drug‐related mortality, and most TEAEs were mild to moderate. No new AEs were identified in this research.

It must be acknowledged that our study had several limitations. First, the small sample size in the fruquintinib‐based therapy is a notable constraint given the vast array of over 70 histological subtypes of sarcoma. Consequently, the results may lack generalizability and impede identifying the optimal population subgroup for fruquintinib treatment. Second, it is a single‐center with retrospectively analysis of routine real‐world data, requiring verification by prospective studies on the safety and efficacy of fruquintinib therapy. Lastly, despite observing encouraging results, the study did not investigate the underlying molecular biology processes of the disease, which warrants further exploration. However, additional investigations are required to further validate these observations.

## 5 CONCLUSIONS

The findings of this study indicate that fruquintinib‐based therapy possesses favorable anti‐tumor properties and acceptable tolerable toxicity in patients with advanced or metastatic sarcoma.

## AUTHOR CONTRIBUTIONS

**Chenliang Zhou:** Funding acquisition (equal); resources (equal); software (equal); supervision (equal); validation (equal); visualization (equal); writing – original draft (equal). **Guowei Qian:** Resources (equal); software (equal); supervision (equal); validation (equal); visualization (equal). **Yonggang Wang:** Resources (equal); software (equal); supervision (equal); validation (equal); visualization (equal). **Hongtao Li:** Resources (equal); software (equal); supervision (equal); validation (equal); visualization (equal). **Zan Shen:** Data curation (equal); formal analysis (equal); investigation (equal); methodology (equal). **Shuier Zheng:** Conceptualization (lead); funding acquisition (lead); project administration (lead); writing – review and editing (lead).

## FUNDING INFORMATION

This research was funded by the Shanghai Pujiang Program (grant number 21PJD050); the National Natural Science Foundation of China (grant numbers 82272835); the Retrospective Clinical Study of the Shanghai Sixth People's Hospital Affiliated to Shanghai Jiao Tong University School of Medicine (grant numbers ynhg202110).

## CONFLICT OF INTEREST STATEMENT

The authors declare no conflict of interest.

## ETHICS STATEMENT

This study was performed according to the Declaration of Helsinki. It was authorized by the Institutional Review Board of Shanghai Sixth People's Hospital Affiliated to Shanghai Jiao Tong University School of Medicine (2022‐KY‐088K), and informed consent was obtained from all patients.

## Data Availability

Datasets are available from the corresponding author upon reasonable request.
